# Quiet Habits: Long Life

**Published:** 1889-09-21

**Authors:** 


### The Doctor's Pulpit.

### QUIET HABITS: LONG LIFE.

Practical teachers—that is, teachers whose minds and consciences will not let them be quiet without results—have great faith in object lessons. Reasoning and argumentation often fail to persuade, because the best argument in the world may be met by a counter-argument; and so great is the natural inconsequence of the average human mind that the counter-argument, though it may be the worst in the world, and of no moment whatever, will be held by many moderately sane people to be quite a sufficient reason for at least suspending judgment. With an object lesson the case is different. If a drunken man fall and break his leg, the eloquence of the temperance advocate is pointed by an undeniable and most significant fact. No counter-argument can disprove the broken leg, and certainly no logic of any kind can cure it except the logic of "splints and rest." Our brief sermon on "Quiet Habits and Long Life" is suggested by personal knowledge of a considerable number of object lessons in the shape of Quakers or members of the Society of Friends.

It is quite true that many "Friends" live long. It is equally true that certain circumstances in their history militate against long life. Among these latter, intermarriage is perhaps the most important of all. The followers of George Fox have never been very numerous, but until late years they have been extremely exclusive. The inevitable result of that has been extensive intermarriage throughout the whole community. The consequences of the frequency of intermarriage have been, and are still, very evident. Quakers as a class are not muscularly robust; many of them are decidedly anæmic, and not a few are mentally feeble. Yet in spite of these practical and serious drawbacks, the Friends, as a class, do more than their proportion of the world's serious business, and they manage to attain to a high average of longevity.

Now this is exactly the kind of fact that true medical science likes to get hold of, and to interrogate and learn from. What is the reason, asks the sensible man, why Quakers, with so many undoubted disadvantages, attain to such a high average of success in all that constitutes worthy life, and also succeed in enjoying their success to an exceptionally old age? The reason, we are convinced, is to be found in their quiet habits and disciplined life. An ordinary doctor, or even layman, would probably have felt much more interest in the subject at this point if we had been able to affirm that the Quakers owed their success and long life to certain drugs, as, for example, to arsenic, phosphorus, strychnia, and the like; or to certain methods of feeding, as vegetarianism, or meat eating, or fruit eating, or wine drinking, or teetotalism, or smoking, and so on. But we submit that that shows a want of real mental capacity. For what, after all, is the true importance of the subject? Does it not consist in the undoubted character of the results? The results are really the things to be considered. As a matter of fact the Quakers are successful in life. As a matter of fact they do live long. Then surely true science will not curl the lip of scorn because these results are obtained by what may be called "natural and simple" processes, instead of by elaborate preparations and out of the way methods.

Our protest is in favour of any and every method which produces the best results, whether the method be of the simple and natural kind, and as old as the world, or brand new from the physiological or the chemical laboratory, or the electrician's legerdemain. The results in the Quaker case, we insist are undoubted, and the methods which produce them must have the credit they deserve.

It is not easy to imagine any greater contrast than that between the quiet habits of the old-fashioned "Friend," and the fussiness, the fret, and the futility of modern ways. Some people try to cram all their past lives into the single day they are at the moment passing through, and all their future lives too. Nothing with them is ever done and finished, nothing is left to the future to be begun. They try to make of life what Carlyle has called "a universal here," and an "everlasting now." The Quaker is trained from childhood to avoid "fussiness," to eschew "fret." The fussy man may be strong, but he is not the strongest. He wears his brains away with superfluous attrition. He cannot know what happiness means. That keenest of pleasures to the masculine spirit, the *otium cum dignitate*, is to him an absolutely incomprehensible mystery. "Dignity" he imagines he knows something about, but "ease" he regards as an abomination of the devil. He has not time to learn to be a man; he prefers to be like a rudderless boat in the open sea, tossed about by every changing wind and restless wave, and steering nowhere.

No human nervous system can stand more than a certain amount of fret. The brain and body that would la t till eighty if well used will probably not endure longer than seventy if over-fretted. Perhaps, in some cases, if the fret is extreme, sixty, or even fifty, may be the utmost limit reached. In a life so short as seventy years is it worth while to throw away twenty by bad management? For that is what it comes to. If a millionaire were dying at fifty how willingly he would give the doctor half of his million if he could make him live with certainty for twenty years longer! And yet, what is impossible for the doctor to do now was probably a very easy task for the patient himself, if he had undertaken it early enough in life.

A quiet life does not mean an idle or a self-indulgent life. Quiet habits are not the peculiarity of the snail only; and even if they were, the snail can reach the top of the garden-wall and let himself down on the inside of the enclosure without difficulty, whilst the more active rat or weasel must remain outside. Quiet habits mean habits of discipline, self-control, orderliness. The fussy person is deficient in the artistic sense as well as in the moral. One cannot even be completely "utter" without practising quiet and order. In the long run it is a question of brains, of real intellectual capacity. If a person has not wit enough to see clearly the advantage of a firm, steady march through the long campaign of life he is not likely to try to learn well the marching part of his drill. He prefers to run when he likes and to dawdle when he likes rather than to move forward in ordered line. In that case he is no soldier, but merely one of the untrained and undisciplined mob; and the chances are a hundred to one that he will be left behind or picked off as a straggler before the campaign is half done. If the Quaker, in spite of intermarriages and consequent anæmia, muscular debility, and some want of mental robustness, can attain to a life both longer and more successful than ordinary merely by the quietness and discipline of his habitual conduct, how much longer and more successful still might be the life history of the ordinary man, who is so superior to the Quaker in physical and mental physique.

